# Assessing and Managing the Current and Future Pest Risk from Water Hyacinth, (*Eichhornia crassipes*), an Invasive Aquatic Plant Threatening the Environment and Water Security

**DOI:** 10.1371/journal.pone.0120054

**Published:** 2016-08-11

**Authors:** Darren J. Kriticos, Sarah Brunel

**Affiliations:** 1 CSIRO, GPO Box 1700, Canberra, ACT, Australia; 2 European and Mediterranean Plant Protection Organisation, 21 Boulevard Richard Lenoir, 75011, Paris, France; Universidade da Coruna, SPAIN

## Abstract

Understanding and managing the biological invasion threats posed by aquatic plants under current and future climates is a growing challenge for biosecurity and land management agencies worldwide. *Eichhornia crassipes* is one of the world’s worst aquatic weeds. Presently, it threatens aquatic ecosystems, and hinders the management and delivery of freshwater services in both developed and developing parts of the world. A niche model was fitted using CLIMEX, to estimate the potential distribution of *E*. *crassipes* under historical and future climate scenarios. Under two future greenhouse gas emission scenarios for 2080 simulated with three Global Climate Models, the area with a favourable temperature regime appears set to shift polewards. The greatest potential for future range expansion lies in Europe. Elsewhere in the northern hemisphere temperature gradients are too steep for significant geographical range expansion under the climate scenarios explored here. In the Southern Hemisphere, the southern range boundary for *E*. *crassipes* is set to expand southwards in Argentina, Australia and New Zealand; under current climate conditions it is already able to invade the southern limits of Africa. The opportunity exists to prevent its spread into the islands of Tasmania in Australia and the South Island of New Zealand, both of which depend upon hydroelectric facilities that would be threatened by the presence of *E*. *crassipes*. In Europe, efforts to slow or stop the spread of *E*. *crassipes* will face the challenge of limited internal biosecurity capacity. The modelling technique demonstrated here is the first application of niche modelling for an aquatic weed under historical and projected future climates. It provides biosecurity agencies with a spatial tool to foresee and manage the emerging invasion threats in a manner that can be included in the international standard for pest risk assessments. It should also support more detailed local and regional management.

## Introduction

The likely impacts of climate change on biodiversity, ecosystem functioning and biological invasions have increasingly attracted the attention of researchers. Sutherst *et al*. [1] and Capdevila-Arguelles and Zilletti [2], among others, describe the likely effects of climate change on invasive alien species (IAS) and their consequent impacts on biodiversity and production systems. The projected shifts in species ranges will provide biosecurity agencies with a moving target in terms of the geographic origins of potential pest species, the pathways by which they could arrive, and the susceptibility of the potentially invaded region. Biosecurity agencies and land managers need to identify as early as possible species and source regions that pose emerging threats, so that they can formulate efficient and effective strategies to manage the emerging threats appropriately.

Under the International Plant Protection Convention, the risks posed by invasive alien species are assessed using a Pest Risk Analysis (PRA) method [3]. Pest risk analysis consists of a framework for organizing biological and other scientific and economic information in order to assess risk, and is the technical justification for international preventative measures on invasive alien species under IPPC [4]. The European and Mediterranean Plant Protection Organization has developed a practical decision-support scheme for pest risk analysis in line with the IPPC recommendations [5]. The PRA framework considers various factors that indicate the likelihood of entry, establishment, spread and impact. Climate change may alter each of these invasion risk factors through both direct (e.g., changing the potential geographic distribution of IAS) or indirect (e.g., shifting agricultural production affecting the likelihood of impact) means. Given the potential for climate change to alter the risks posed by IAS to a jurisdiction, the effects of projected climate changes should logically be integrated into PRAs, although there are as yet no standard methods for doing so.

Some attention has been applied to understanding the likely impacts of climate change on the potential distribution of a variety of invasive plant species such as trees [6,7], woody shrubs [8], grasses [9] and lianas [10]. However, as far as we know there have been no published examples of attempts to assess the impacts of climate change on the potential distribution of an aquatic plant species. A CLIMEX projection for *Eichhornia crassipes* has been undertaken EPPO PRA framework, to analyse the risk this species poses to European and Mediterranean countries.

Water Hyacinth, *Eichhornia crassipes* (Pontederiaceae) (Martius) Solms-Laub. is one of the world’s worst weeds [11]. It is an erect floating herb that reproduces by seed, or from stolons [12]. It originates from Amazonia, Brazil [13] and probably Argentina [14], with anthropogenic spread to other areas such as Venezuela, parts of central South America, and the larger Caribbean islands [15,16].

*Eichhornia crassipes* is used as an ornamental plant in garden ponds. This attribute has certainly helped its spread. However, its aquatic habit and potentially explosive growth rate lead to it choking waterways and water supply equipment. *Eichhornia crassipes* forms dense mats that reduce light transmission to submerged plants and competes with other plants, often displacing wildlife forage and habitats [17], depletes oxygen in aquatic communities [18] resulting in a lack of phytoplankton [19] and an alteration of the composition of invertebrate communities [20,21], ultimately affecting fisheries. It reduces the area available for water birds, and harbours mosquitoes and other animal disease vectors [22]. *Eichhornia crassipes* increases evapotranspirative water losses, with estimates varying from 2.67 times [23] to 3.2 times [15] more from a mat of *E*. *crassipes* compared to open water. This effect is of particular relevance in regions that suffer chronic or seasonal droughts (e.g., Mediterranean or Wet-Dry tropics). In Spain and Portugal as in other parts of the world, impacts have been noted in fisheries, recreational water sport and boat navigation [24,25,26]. Such impacts would be particularly detrimental in vulnerable countries whose economies rely for a substantial part on tourism (e.g. Tunisia).

*Eichhornia crassipes* is now present on all continents except Antarctica, and has invaded all tropical and sub-tropical countries [22,27], as well as some parts of the Mediterranean basin. It is considered one of the world’s most invasive aquatic plants and considerable effort is expended worldwide to manage *E*. c*rassipes* and its impacts on agriculture, the environment and human activities [28,29]. Legislation to prevent the plant from being sold or distributed is presently being developed in the USA, Australia, New Zealand, South Africa, the United Kingdom, Morocco, and Portugal [29]. After completion and approval of the EPPO PRA on *E*. *crassipes*, in 2008 the species was recommended for regulation within European and Mediterranean countries.

CLIMEX [30,31] is a process-oriented climate-based niche modelling package. It enables users to project the climatic potential distribution of poikilothermal organisms based primarily on their current distribution. It has been widely used to model the potential distribution of many invasive weeds [6,10,32,33], insect pests [34,35,36,37], and plant diseases [38,39,40,41].

CLIMEX is unique amongst climate based niche modelling packages in that a combined inductive-deductive method can be used to create models for estimating species’ potential distributions. It uses climatic growth response functions that obey Shelford’s Law of Tolerance [42], which indicates that a species growth response to a given environmental resource has an optimal value, within an upper and lower bound. These growth indices are combined in accordance with the Sprengel-Liebig Law of the Minimum, which holds that the resource in shortest supply provides the greatest limitation on the growth of a species [43]. By employing biologically relevant climate response functions, and fitting parameters to presence-only data, CLIMEX provides an insight into the taxon’s ecological response to climate. Where additional information such as phenological or direct experimental observations of species responses to climatic variables is available, these data can be used to inform parameter choices or confirm the biological plausability of choices. This approach can provide a robust cross-validation of model parameters by considering information from across multiple information domains.

CLIMEX includes mechanisms to explore the sensitivity of a species range and growth dynamics to climate change. Because of its process-oriented formulation it satisfies the requirement identified by Thuiller [44] for niche models to capture the full species response curve for relevant environmental variables in order to confidently project climate change responses. It is therefore able to be applied to novel climate situations with more confidence than regression-based or climate matching methods that have been shown to give erratic, unreliable results when applied to novel climate situations [44,45,46,47]. In this paper a species niche model of the climate responses of *E*. *crassipes* was developed in order to: (i) project the climatic potential distribution and relative abundance of *E*. *crassipes* throughout the world, identifying areas of climatically suitable land that are potentially, but as yet uninvaded, (ii) apply global climate scenario to assess the sensitivity of this potential distribution to climate change, and (iii) suggest biosecurity management options to mitigate the identified historical and emerging invasion risks.

## Methods

### Climex

CLIMEX [30,31] was used to fit a Compare Locations model to the distribution records for *E*. *crassipes*. In the CLIMEX Compare Locations module, selected climate response functions for population growth and stress are usually manually adjusted until a satisfactory agreement is reached between the modelled and known distribution of the study organism in the native range, and any introduced ranges besides those for which the model is needed [48,49]. This latter consideration is an attempt to address issues of climatic range expansion that are sometimes noted when species are introduced to locations outside of their native range [36,48,50], and when exploring issues of climate change [51].

CLIMEX uses a set of fitted growth and stress functions to assess the potential for a species to persist and grow at each location for which relevant climatic data are available. CLIMEX calculates an overall annual index of climate suitability, the Ecoclimatic Index (EI), which is theoretically scaled between 0 (unsuitable) and 100 (climatically perfect all year round). In practice, a score of 100 is rarely achieved, and then only in locations with high climatic stability such as for species that inhabit some equatorial regions. The EI represents the net effect of the opportunity for growth as indicated by the annual Growth Index (GI_A_), discounted by the Stress Index (SI) and the interaction Stress Index (SX) (Eqs 1–4).

$$EI=GI_{A}\times SI\times SX$$ (1)

$$GI_{A}=100\sum_{i=1}^{52}TGI_{W_{i}/}/52$$ (2)

$$SI=\left(1-\frac{CS}{100}\right)\left(1-\frac{DS}{100}\right)\left(1-\frac{HS}{100}\right)\left(1-\frac{WS}{100}\right)$$ (3)

$$SX=\left(1-\frac{CDX}{100}\right)\left(1-\frac{CWX}{100}\right)\left(1-\frac{HDX}{100}\right)\left(1-\frac{HWX}{100}\right)$$ (4)

Where CS, DS, HS, and WS are the annual cold, dry, heat and wet stress indices respectively, and CDX, CWX, HDX and HWX are the annual cold-dry, cold-wet, hot-dry and hot-wet Stress Interaction indices. In addition to the growth and stress indices, it is possible to add additional requirements for species persistence such as an obligate or facultative diapause, or a minimum annual heat sum required to complete a generation. The weekly growth index GI_W_ is composed of a separate soil moisture index (MI) and a temperature Index (TI), which are formulated using three-segment linear equations, varying between 0 (no growth) and 1 (optimal growth) to comply with Shelford’s Law of Tolerance. By combining MI and TI together multiplicatively, GI_W_ and its annual integral GI_A_ satisfy the Sprengel-Liebig Law of the Minimum.

When assessing the goodness of fit of CLIMEX models for invasive species it is important to weight apparent omission and commission errors differently [52]. Because of the potential financial or ecological consequences of underestimating a species’ invasive potential compared to the costs of additional unnecessary surveillance, apparent commission errors are to be preferred to omission errors. We use the term *apparent* errors here because for invasive species the “truth” is usually either unknown or impossible to define. Therefore, during model calibration, the parameters are adjusted so as to try to simultaneously ensure that all known presence points are projected to lie in climatically suitable locations, and that the ecophysiological model parameters are biologically reasonable, satisfying all known sources of relevant niche-defining information for the target species. Where inconsistencies are identified, all information sources are questioned, and decisions regarding the resolution of the confrontation are documented.

### Climate data

Two sets of climate data were used in this project. The *E*. *crassipes* model was fitted using the 1961–1990 climate normals derived from the CRU 0.5 degree gridded dataset CL1.0 [53]. The reformatting and derivation of temperature and relative humidity variables from this dataset for use in CLIMEX is described in [37]. This dataset was used to project the potential distribution of *E*. *crassipes* under current climate conditions.

The second set of climate data were derived from Global Climate Model’s (GCM’s) projecting future climatic conditions. The GCM data employed in this project were drawn from the World Climate Research Programme's (WCRP's) Coupled Model Intercomparison Project phase 3 (CMIP3) multi-model dataset [54]. Three models were selected because they satisfied the following criteria: (i) monthly means of daily minimum and maximum temperature, monthly rainfall total, and monthly surface level specific humidity for the A1B and A2 emissions scenarios were available, (ii) the models had a relatively small horizontal grid spacing, and (iii) the models had to have a high skill score [55] in representing the observed climate between 1980 and 1999 [56].

In recognition of the observations by Rahmstorf *et al*. [57] and Manning *et al*. [58] that recent global historical temperature and emissions observations indicate that global temperatures are tracking “hot”, only the higher emissions scenarios (the “A” family) are included in this project, rather than the more conservative “B” family of scenarios. The three GCMs that best met the selection criteria are: (i) CSIRO Mark 3.0 (CSIRO, Australia), (ii) NCAR–CCSM (National Centre for Atmospheric Research, USA), and (iii) MIROC—H (Centre for Climate Research, Japan). Data from these GCMs were pattern-scaled to develop individual change scenarios for the year 2080 relative to the base climatology [59]. The three GCMs also cover a range of climate sensitivity i.e., the amount of global warming for a doubling of the atmospheric CO_2_ concentration compared with 1990 levels (CSIRO Mark 3.0, 2.11°C, NCAR–CCSM, 2.47°C and MIROC-H, 4.13°C). The GCM data were transformed in a similar manner to that described by Stephens *et al*. [37] to provide the required change factors for each of the variables of monthly averages for daily minimum temperature, maximum temperature, relative humidity at 9 am, and 3 pm, and monthly totals for precipitation.

### Present distribution of *Eichhornia crassipes*

**Fig 1:**
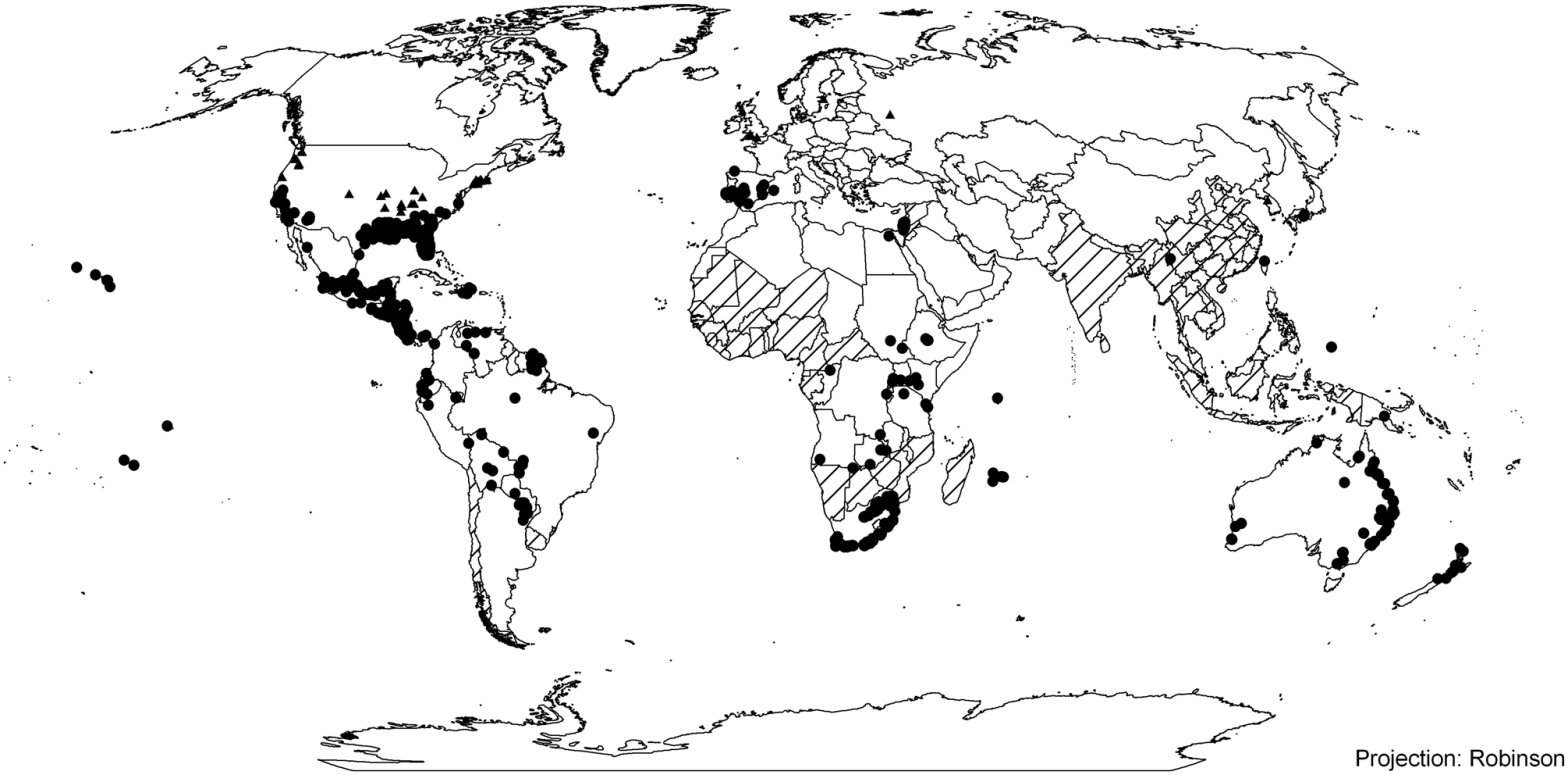
The global distribution of *Eichhornia crassipes*, including established and casual populations. Where information has been provided by country, these administrative areas have been shaded. Where more precise distribution data is available this is indicated as dots, with established population indicated as circles, and ephemeral populations as triangles. Source: various (see Acknowledgements)

The global distribution of *E*. *crassipes* was assembled from a large number of sources in the framework of the EPPO PRA on the species ([28]; Fig 1). *Eichhornia crassipes* is distributed throughout the world, flourishing in tropical and subtropical regions, extending into Mediterranean climatic areas. The latitudinal limits of its distribution were considered to be 40° [27], until it expanded its northern distribution in Spain (Fig 1). A worldwide updated distribution of *E*. *crassipes* is maintained by EPPO in the EPPO Plant Quarantine Data Retrieval system (see www.eppo.int/DATABASES/pqr/pqr.hrm.

According to [14], *E*. *crassipes* is native to Argentina. Whilst it is present in La Rioja but not in Formosa or Salta, the CLIMEX projections indicate that the climate appears favourable for *E*. *crassipes* in both Formosa and Salta. According to a CLIMEX Match Climates analysis, the climate in these two cities is similar to parts of South Africa and the eastern coast of Australia where the species is present and invasive (data not shown). There is therefore no apparent climatic reason why the species would be absent from this area of its native range. It is therefore assumed that this is missing information from Argentina, or that there is some biotic or other non-climatic factor responsible for its absence there.

**Fig 2:**
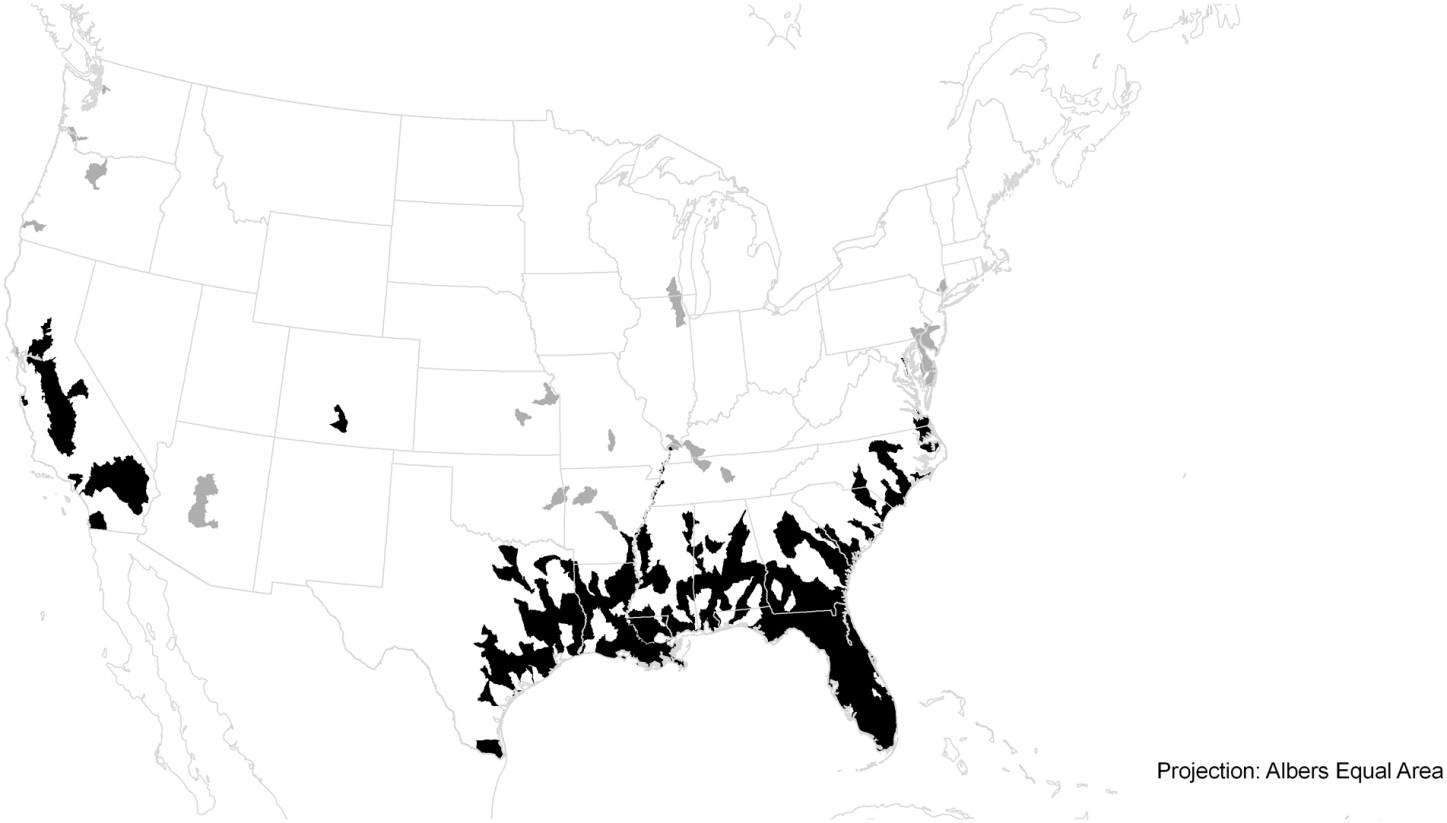
United States of America showing the casual and established population range of *Eichhornia crassipes*. Black areas indicate established populations, grey areas indicate ephemeral or casual populations that die out each winter. The mapping unit is the USGS 8-digit Hydrologic Unit Catchment. Source Pam Fuller, USGS, pers. comm. The outlying established population in Colorado is in a geothermal spring with a consistent temperature of approximately 31°C (Amy Benson, USGS, pers. comm., 2009)

CLIMEX models fitted to field data are sensitive to whether the species is ephemeral (= casual or transient) or naturalized (= established) at each site [60]. In the USA, records in Colorado, Connecticut, Illinois, Maryland and New England corresponded to observations where *E*. *crassipes* is either casual and frequently introduced, or persisting in favourable microhabitats such as in geothermal pools (Fig 2). The species is also a casual in Seattle (Washington State, Sauter Messick, pers. comm., 2008), the British Isles [61] and in Moscow, dying due to cold stress during winter. In addition, the species is cultivated in botanic gardens in Amsterdam (The Netherlands), Colonia (Germany), Brno (Czech Republic) and Slovak Republic, but does not thrive outdoors in these locations. These data therefore played a limited role in fitting or validating the modelled potential distribution of *E*. *crassipes*. When assessing model performance, in these areas we assumed that the conditions would be suitable for growth during part of the year, but otherwise unsuitable for long-term persistence (GI_A_ > 0 and EI = 0).

### Phenology and Environment

*Eichhornia crassipes* is a free-floating aquatic macrophyte, reproducing both vegetatively, via ramets formed from axillary buds on stolons, and sexually through seed production [12]. *Eichhornia crassipes* flowers year-round in mild climates. *Eichhornia crassipes* colonises still or slow moving water. It forms thick mats in estuarine habitats, lakes, urban areas, watercourses, and wetlands. It can tolerate extremes of water level fluctuation and seasonal variations in flow velocity, as well as extremes of nutrient availability, pH, temperature and toxic substances [27], but does not tolerate brackish and saline water [62]. For a given temperature, growth is directly correlated with nutrient concentrations [27], as N and P increase in concentration [63,64].

### Influence of climatic factors on distribution

#### Rainfall

Being aquatic, *E*. *crassipes* is critically dependent upon the presence of standing water. As the presence of standing water is a function of precipitation, evaporation, meso-topography and human practices, we decided to treat the presence of standing water separately from the other temperature-related climatic factors.

#### Temperature

*Eichhornia crassipes* is reported to be winter hardy, but sensitive to frost. Frosts kill the leaves and upper petioles that protect the rhizome, but prolonged cold temperatures, below 5°C, may also kill the rhizome, resulting in death of the plants [65]. The ability of the seeds and bulbs to survive winter conditions is likely to be another range-limiting factor. In the United States, rhizomes and seeds can survive winter in water that is covered in ice, where temperatures in the sediment do not fall too low (John Madsen, Mississippi State University, pers. comm., 2009). For *E*. *crassipes* to persist in seasonally cool climates where the leaves and upper petioles are killed during winter, it is also likely to need a minimum heat sum (length of growing season) necessary to regrow and mature (produce seeds) prior to the return of damaging cold conditions. This suggests that there may be two separate cold stress functions operating on the populations at the cold range margins: one associated with the damaging low temperatures, and an energetic balance mechanism associated with insufficient daily maximum temperatures to allow photosynthesis to offset respiratory losses.

### Fitting parameters

To fit the Compare Locations models the growth and stress parameters are iteratively adjusted until 1) the region where EI > 0 encompasses all location points for the taxa, using the minimum area to do so, 2) all parameters are biologically reasonable, and 3) all stress functions are either necessary to achieve a satisfactory fit, or are strongly supported by information from other knowledge domains (e.g., observations of frost intolerance, or measured annual heat sums to complete a generation). The parameters used in the CLIMEX model for *E*. *crassipes* are summarized in Table 1. The role and meaning of these parameters are fully described in Sutherst *et al*. [66], and their values are discussed below. It should be noted that the meteorological data used in this model represent long-term monthly averages, not daily values. This means that it is not always possible to compare directly values derived using the model fitted to distribution data using long-term climate averages with instantaneous values derived through direct observations. This issue appears to apply mostly to stress parameters relating to maximum and minimum temperatures. For the growth parameters, the averages probably accord sufficiently well under most circumstances for direct observation of growth responses to temperature to be applied to the model directly.

**Table 1:** CLIMEX parameter values used for *Eichhornia crassipes*. Parameter mnemonics taken from [31].

*Index*	*Parameter*	*Value*^a^
Temperature	DV0 = lower threshold	12°C
	DV1 = lower optimum temperature	25°C
	DV2 = upper optimum temperature	30°C
	DV3 = upper threshold	34°C
Cold stress	TTCS = temperature threshold	0.5°C
	THCS = stress accumulation rate	-0.01 Week^-1^
	DTCS = heat sum temperature threshold above DV0	10°C Days
	DHCS = stress accumulation rate	-0.00035 Week^-1^
Heat stress	TTHS = temperature threshold	37°C
	THHS = stress accumulation rate	0.001 Week^-1^

^a^Values without units are a dimensionless index of a 100 mm single bucket soil moisture profile.

The climatic responses of *E*. *crassipes* (Table 1) were derived by fitting the stress parameters combined with the growth parameters taken from Kasselmann [67] to the known distribution in its native and selected exotic locations (Africa and the USA), and then comparing the projected and known distributions within Europe and Australia as a means of validating the model.

#### Stress indices

In CLIMEX, stress indices indicate negative population growth potential and vary between 0 and ∞, where a value of 100 or greater indicates lethal conditions. When threshold conditions are exceeded, stresses accumulate on a compounding weekly basis. The thresholds and accumulation rates are user-defined parameters. Wet and Dry stresses were ignored for the *E*. *crassipes* model since it is aquatic.

#### Heat stress

The plant is present in Mali and Niger where maximum temperatures are very high. According to Kasselmann [67], the species has a maximum growth temperature (DV3) of 33–35°C. Similarly, according to [65], growth is retarded above 34°C. The heat stress threshold was set to 37°C so as to fit known distribution records. It is assumed that the stress accumulates moderately rapidly above this threshold, and the rate was set to -0.001 week^-1^ (THHS).

#### Cold stress

The reported frost sensitivity of *E*. *crassipes* suggests that a cold stress temperature model might be appropriate. In order to fit the known northern range limit in the USA, the cold stress temperature threshold (TTCS) was set to a weekly average temperature of 0.5°C. This indicates that the species begins to accumulate stress when long-term monthly minimum temperatures drop below 0.5°C, which means that on average that climate station would receive several frost events per week. Since the species has been reported to remain alive at -5°C for a time but then dies, it is assumed that the cold stress accumulates moderately rapidly, and the rate (THCS) is set at -0.01 week^-1^. In order to fit the model to the distribution records in the central USA it was also necessary to include a degree day cold stress. This stress mechanism represents the necessary balance between anabolic and catabolic processes necessary to maintain biomass. The degree day threshold for metabolic maintenance DTCS was set to 10 and the stress accumulation rate (DHCS) was set to -0.0003 week^-1^ since the species is supposed to accumulate this stress slowly.

#### Growth and Temperature indices

The Growth Index simulates how favourable each location is for population growth, and is scaled from 0 to 100. The weekly temperature index values are integrated to give the growth index GI_A_, which is re-scaled from 0 to 100. When the effects of soil moisture are ignored, the Temperature Index (TI) and the GI_A_ values are equal. According to Kasselmann [67], the minimum temperature for growth of *E*. *crassipes is* 12°C, its optimum growth temperature is 25–30°C, and its maximum growth temperature is 33–35°C. Owens and Madsen [65] report that optimal growth occurs at temperatures of 28 to 30°C, while growth ceases when water temperatures drop below 10°C and it is retarded above 34°C. It is assumed that these reported temperatures are water temperatures. Accordingly, the minimum threshold for population growth, DV0, was set to 12°C, the lower and upper temperature for maximum growth rates (DV1 and DV2) were set to 25°C and 30°C respectively. The maximum threshold for population growth (DV3) was set to 34°C, following the same source, and lower than the heat stress threshold.

#### PDD

In the interests of parsimony a minimum annual heat-sum for survival (PDD) was not used in this model. Under favourable conditions the plant can produce seeds and reproductive vegetative parts within 12 weeks from germination [68]. However, there was nowhere within its known range where the distribution appeared to need this requirement to constrain it. That is, the need for a minimum annual heat sum is met in all locations where the cold stress is not lethal. If the length of thermal time taken for the plant to mature was available at a series of sites where the annual temperature profile was known, then this parameter could be estimated with confidence.

## Results

### Current Climate

**Fig 3:**
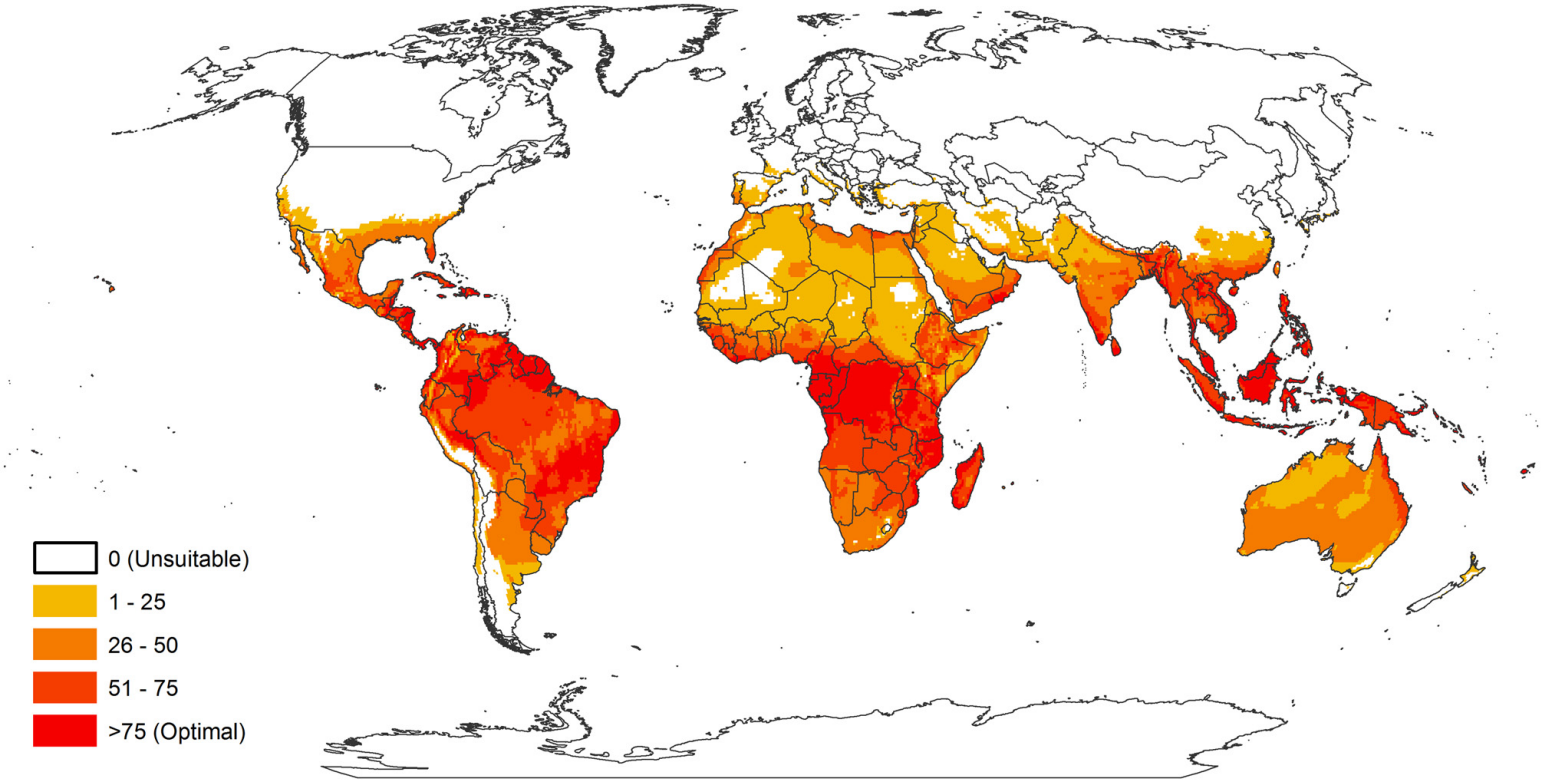
World map showing climate suitability for *Eichhornia crassipes* under current climate modelled using CLIMEX. It is assumed that *Eichhornia crassipes* will always be restricted to waterways within this suitable temperature envelope.

**Fig 4:**
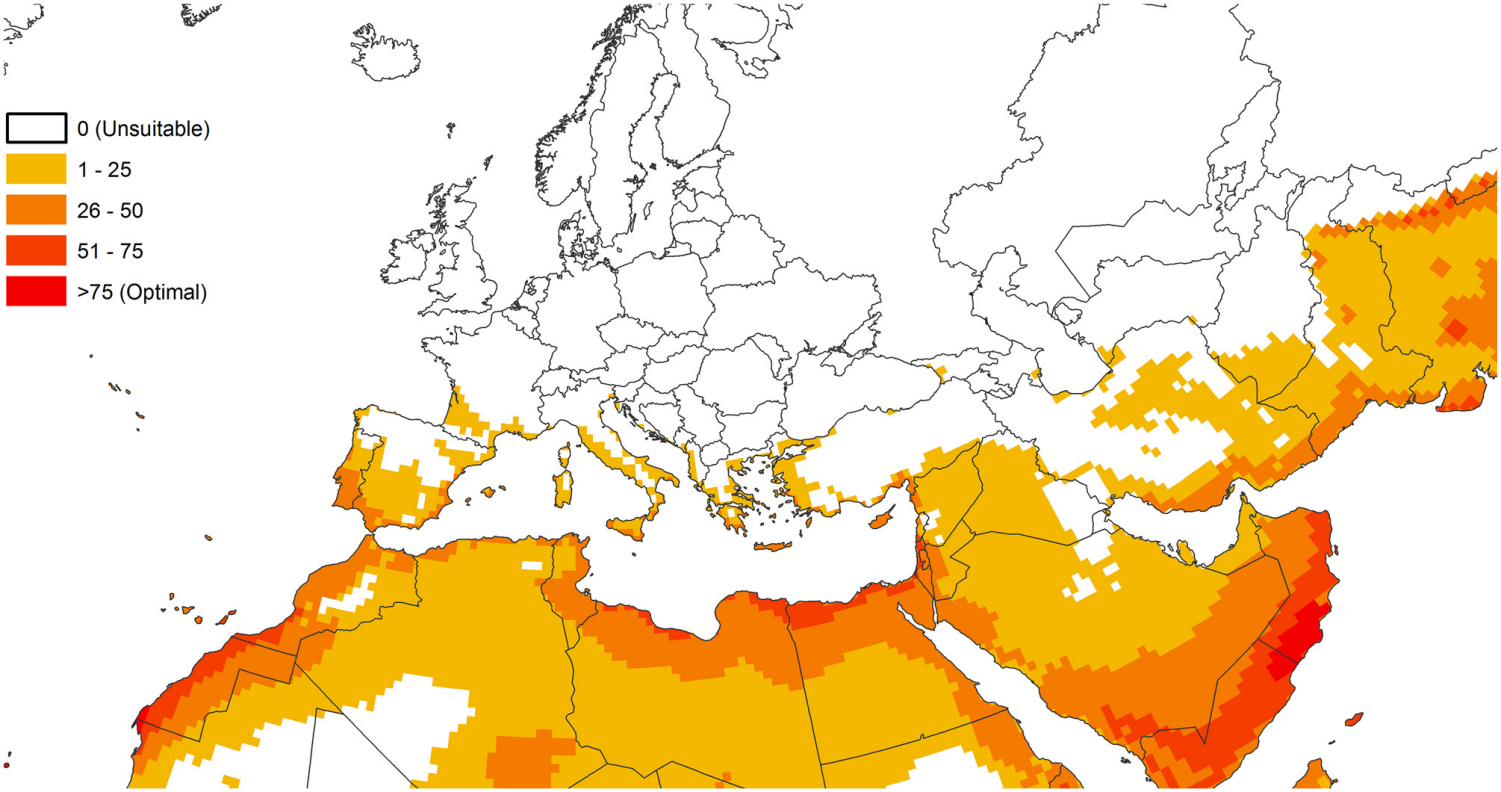
Modelled potential distribution of *Eichhornia crassipes* under current climate conditions in European and Mediterranean countries. It is assumed that *Eichhornia crassipes* will always be restricted to waterways within this climatically suitable envelope.

The areas estimated to be climatically suitable for *E*. *crassipes* under current climatic conditions are illustrated for the world (Fig 3). The projected climate suitability for *E*. *crassipes* as indicated by a positive value for the Ecoclimatic Index is entirely consistent with the current known distribution of sites and administrative regions in which *E*. *crassipes* has established populations (compare Figs 1 and 3). The potential geographical range of *E*. *crassipes* is extremely broad. In general terms its poleward extent is bounded by cold stress, and its ability to inhabit some tropical areas in Africa is limited by heat stress. Within these broad geographic limits, some high altitude areas are also too cold for persistence. Throughout this region of broad climatic suitability (Fig 4), *E*. *crassipes* is only likely to pose an invasion threat where there are sources of standing water.

In the native range of *E*. *crassipes*, the climatic potential range appears to extend into cooler areas of Argentina than it has been reported. This suggests under-reporting, some form of competitive exclusion or other biotic effect, or unfavourable land management in this region.

In central and north America, the potential distribution accords well with the known distribution. In the arid areas of northern Mexico and northern Texas it is likely that there are few suitable sources of standing water to support *E*. *crassipes*. The ephemeral populations in North America (Fig 2) occur in locations where the CLIMEX model indicates a favourable annual Growth Index, GI_A_, but an unsuitable Ecoclimatic Index.

In central and southern Africa, the plant seems to be reported throughout much of the climatically suitable range, particularly where there are large lakes e.g. Malawi [69], or irrigation infrastructure e.g. South Africa [70]. Heat stress appears to limit the spread of *E*. *crassipes* in parts of Central Africa such as in Mali (Araouane), South of Algeria (Oualen Bordj) and Sudan (Merowe, Dongola). Cold stress limits the range of *E*. *crassipes* in a few small high elevation areas in South Africa.

The northern boundary of the potential distribution in Europe is clearly defined by cold stress, with climatically suitable habitat encircling the Pyrenees and the Massif Central in southern France. All of the countries of the Mediterranean basin are at significant risk, with Libya, Egypt, Israel, and Lebanon at most risk in terms of area and degree of climate suitability (Fig 4). Nonetheless, the standing water resources throughout the Mediterranean basin are critical to human survival as a means of surviving the summer drought period that typifies the Mediterranean climate.

In Spain, *E*. *crassipes* has been recorded in Laguna de Arnao, Castrillon (43°32’N, 7°01’W), an area that is modelled as being climatically too cold for its persistence. However, this site has been reported to be protected (Ruiz Tellez, pers. comm., 2008), perhaps explaining this anomaly.

In Sardinia, the invasion by *E*. *crassipes* became evident in 2010 when the river Mare ‘e Foghe in the Oristano Province (39°59’N, 8°32’E) was covered for 8 km over a surface of 560 000 m^2^. The species prevented recreational activities and in particular fishing competition [71]. The projected distribution fits with this record. The projected distribution also fits with a latest outbreak discovered in the south of Turkey on the Asi River which runs from Lebanon, through Syria to Turkey [72].

### Future Climates

**Fig 5:**
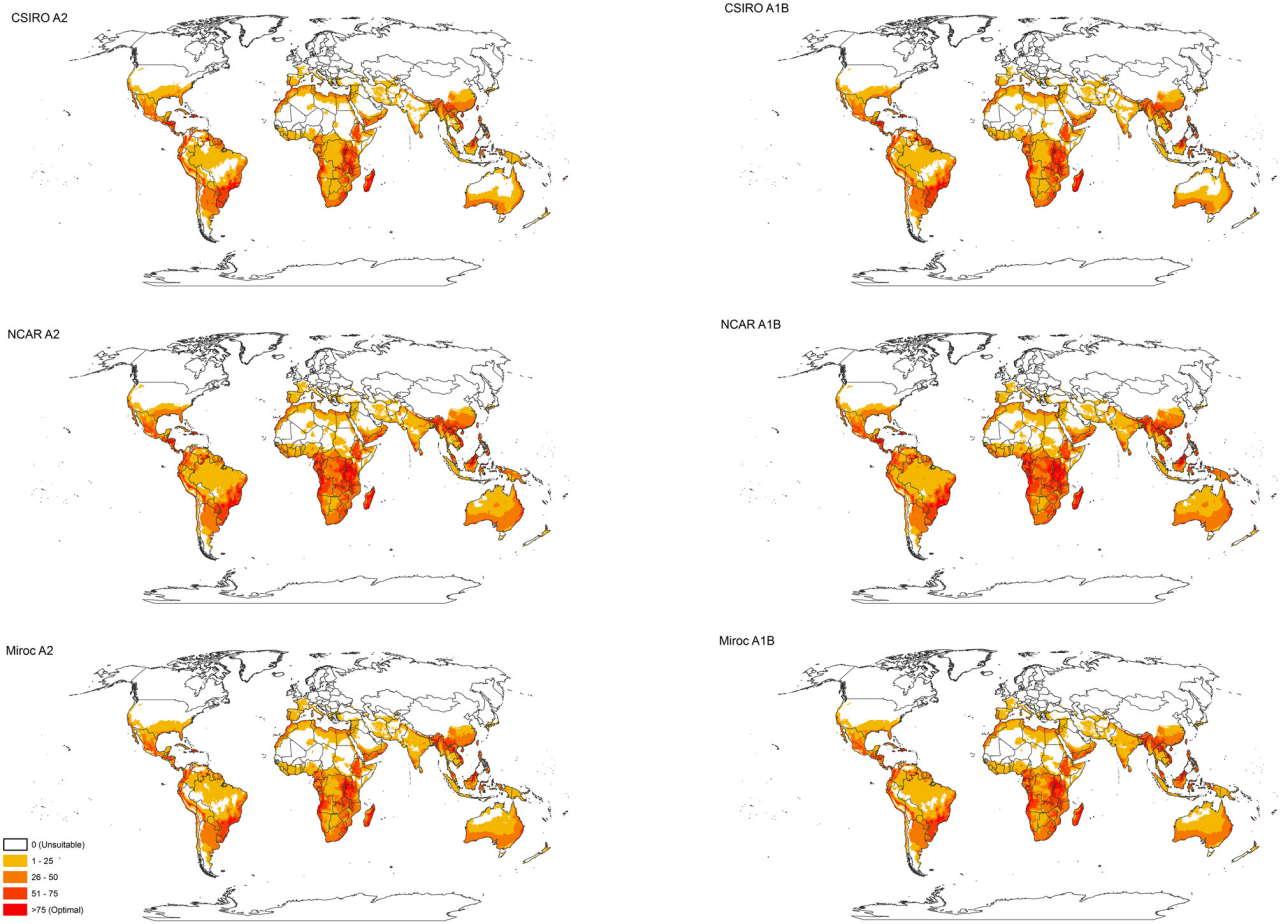
Modelled global potential distribution of *Eichhornia crassipes* using climate scenarios for 2080 under the A2 and A1B SRES emissions scenarios. The global climate models are indicated next to the maps.

The overall impact of projected climate changes by 2080 upon the potential distribution of *E*. *crassipes* in the northern hemisphere will be to allow it to expand its range northwards as cold stress limits are overcome. This pattern is most apparent in North America, Europe and north-eastern China (Fig 5). Elsewhere in Asia the cold gradients at the range margins for *E*. *crassipes* appear to be steep, and so there is little opportunity for range expansion. Increasing heat stress is likely to reduce its potential range in Saharan Africa, the Middle East and India, and under some emission scenarios the Amazon Basin and north-western Australia (Fig 5).

In contrast to the northern hemisphere, the southern hemisphere has little land area that is marginally too cold for *E*. *crassipes*. Hence, there is little potential for *E*. *crassipes* to expand its potential range in the southern hemisphere in the future. The mountain ranges of the Andes in southern South America and the highlands of southeastern Australia constitute a steep cold stress gradient, which will afford limited opportunity for range expansion under a warming climate (Fig 5). The low-lying areas of Tasmania in Australia, and the South Island of New Zealand may become suitable to support invasive populations of *E*. *crassipes* in the future (Fig 5). Similarly, the presently unsuitable highlands of South Africa appear to become suitable under a warming climate (Fig 5).

In the future climate scenarios, the variance between the GCMs seems to be greater than the variance between the emissions scenarios (Fig 5). However, it should be noted that the variance between the emissions scenarios certainly does not represent the full uncertainty, but rather some degree of uncertainty around the high emissions scenarios that best accord with observations to date [57].

## Discussion

Under present climatic conditions, *E*. *crassipes* appears to be affecting or threatening a significant proportion of infrastructure for storing and supplying freshwater throughout Central America, southern USA, Africa, southern Europe, the Middle East, southern Asia, and Australasia. Elsewhere, the threats posed by this aquatic plant are possibly most acute in regions that suffer seasonal or chronic drought (e.g., southwestern USA, east central and southern Africa, southern Asia and much of Australia), with increased evapotranspiration losses, disruption of supplies etc.

### Non-climatic distribution factors

In this analysis, we have not attempted to model the availability of aquatic habitat for *E*. *crassipes*, but this has been done by Baker *et al*. [73]. Being climate-based, the CLIMEX model ignores other non-climatic factors that also determine habitat suitability for a particular species. *Eichhornia crassipes* can tolerate pH levels from 4.0 to 10.0, though it prefers pH values of 6 to 8 [25], and is therefore not limited by this factor. *Eichhornia crassipes* also grows best in water that is high in nutrients [25,63,64]. Fertiliser effluent tends to enrich waterways, and it is expected that waters will get richer in nutrients in the future, and become more suitable for *E*. *crassipes* [29]. The effect of this feature of the CLIMEX model will be to underplay the degree of variability of the problem posed in any given water body. If nutrient levels are high, and pH levels are neutral, then *E*. *crassipes* will likely be more problematical than otherwise, for a given temperature regime.

### Climate change

This study highlights some areas at threat of invasion by *E*. *crassipes* under present or possibly future climates, and where it is important to prevent its introduction. It also identifies areas of marginally climatically suitable habitat in which it would be prudent to manage the populations to prevent or minimise future management problems by dealing with them whilst the problems are more manageable.

The future climate scenarios highlight several areas of emerging or increasing threat. In the Euro-Mediterranean region, the following countries are currently at greatest risk: Albania, Algeria, Bosnia and Herzegovina, Croatia, France (including Corsica), Greece, Israel, Italy (including Sardinia, Sicilia), Jordan, Montenegro, Portugal (including Azores and Madeira), Slovenia, Spain (including Baleares and Canary Islands), Turkey and Tunisia. All future climate scenarios explored here indicate that the invasion threat posed to Europe is likely to increase significantly, including most of France, Greece, Italy, Portugal and Spain. At present, *E*. *crassipes* has a very limited distribution in Europe, and it would seem prudent to explore the feasibility of a coordinated eradication campaign or a strategic containment strategy. To this end, EPPO has published National Regulatory Control Measures [74] to provide countries with eradication and containment guidelines for *E*. *crassipes*. Another option to consider in this region if the species attains high populations would be biological control, which has been shown to be effective elsewhere. At this cold range margin however, it may be advisable to use climate-matching techniques such as those described by Robertson *et al*. [75] to prioritise the search for agents in those regions with a similarly cool climate. In the USA, the northern range boundary *E*. *crassipes* is limited by cold temperatures [76]. Under the future climate scenarios explored here, its range could extend considerably further northward. Within climatically suitable regions, increased rainfall and hurricane intensity could result in more frequent and intense flooding events, facilitating *E*. *crassipes’* dispersal [77]. As observed for *Mimulus guttatus* by [78], vegetative fragmentation, as well as distance of spread of the plant through water could be increased with higher water velocities due to unpredictable flood pulses.

### Management

Where *E*. *crassipes* has become established in such proportions that eradication is infeasible, it may be worthwhile exploring opportunities to exploit the infestations as a bioenergy resource. For example, it has been estimated that in Malawi *E*. *crassipes* could contribute 10 MW yr^-1^ to a population that is suffering from a general lack of access to any form of electricity, and the 6% of the population that does have access to electricity suffers from chronic interruptions to supply. Where *E*. *crassipes* has not already established, or can be contained, there is a risk that it could become a conflict species, with the potential for both benefits, and negative impacts. In such situations, the twin goals of utilisation and local eradication may be incompatible, or at least an uncomfortable mix. In Australasia, the islands of Tasmania in Australia, and the South Island of New Zealand presently appear free of *E*. *crassipes*. On the islands that depend critically upon dams for hydroelectricity and drinking water, it would seem prudent to employ a biosecurity strategy designed to use the natural oceanic barrier to help prevent the introduction and establishment of *E*. *crassipes*. The judicious use of legislation preventing the sale or distribution of this plant, along with a public education campaign, and targeted surveillance may provide the ounce of prevention that negates the need for the pound of cure [79]. Whilst it is likely that under current climatic conditions, *E*. *crassipes* would be able to persist in these islands only in protected microhabitats, the risk that these potential invasion foci could pose in the future as the climate becomes more clement for *E*. *crassipes* should be borne in mind because biological invasions are usually irreversible.

In the USA, the distribution of casual and established populations of *E*. *crassipes* has been well mapped by the USGS (Fig 3). The CLIMEX model presented here could be used to define different management regimes throughout the USA, depending upon the climate suitability of different catchments under current and future climates. By combining the current and future climate suitability projections, those catchments that are presently unsuitable for persistence, but are likely to become so in the future could be identified. These marginally suitable catchments could be efficiently targeted for application of a strategy of catchment-wide eradication and hygiene. Addressing this problem on a catchment basis is logical because *E*. *crassipes* can spread vegetatively throughout a catchment. As in Europe, the management of this weed would require suitable legislative changes, public education and compliance management to stop people living in colder climates from redistributing *E*. *crassipes* into more clement states, and to prevent aquarium and pond owners from dumping unwanted specimens into local waterways in these present or emergent climatically suitable areas.

In non-European Mediterranean countries, *E*. *crassipes* should be regulated as in Morocco [80], and surveillance should be established over the territory to eradicate or contain the plant in case of an outbreak. Because the movement of people and goods within Europe is only loosely-regulated, and because Europe spans a wide range of climate suitability for *E*. *crassipes*, effective management of this plant invasion could be particularly problematical. As is apparent in Figs 4 and 5, the area of Europe that is climatically suitable for *E*. *crassipes* invasion is in the south. Throughout northern Europe, conditions are suitable for cultivation of *E*. *crassipes* as an ornamental, either in sheltered conditions, or as a summer casual being replanted each year. Even if trade in *E*. *crassipes* was prohibited in countries at risk in Europe (Croatia, France, Greece, Italy, Spain and Portugal, etc.) and European-wide import bans were implemented, nurseries or gardens in more temperate countries might be a source of re-entry of *E*. *crassipes* into risk areas. For instance *Lysichiton americanus* was thought to have been introduced into France by Dutch people having bought a secondary house in France, introducing new plants that were not traded in France [81]. Under such circumstances, any strategy of local eradication or strategic control would need to consider the effect of potential re-introductions from regions where *E*. *crassipes* is a casual. Unless the benefits of horticultural production in those countries suitable for transient occupation by *E*. *crassipes* were so overwhelmingly positive that the beneficiaries could compensate those countries suffering the impacts of continued re-invasion, an economically optimal solution would be for all the European countries to participate in a coordinated import ban and associated control or eradication campaign. Such questioning is particularly important in the view of implementing the EU regulation on invasive alien species [82]. If *E*. *crassipes* is regulated, the general public (particularly land managers, horticulturists, municipalities and gardeners) would need to be made aware on the threats caused by this species, and materials produced to help the public to recognize it in order to organize an efficient early warning system through citizen science. *Eichhornia crassipes*, being a beautiful ornamental plant easily recognizable and having enormous detrimental impacts represents an excellent candidate for communication campaigns in Europe and in the Mediterranean region.
